# Challenges in abdominal re-exploration for war casualties following on-site abdominal trauma surgery and subsequent delayed arrival to definitive medical care abroad – an unusual scenario

**DOI:** 10.1186/s12873-022-00687-5

**Published:** 2022-07-18

**Authors:** Amitai Bickel, Konstantin Akinichev, Michael Weiss, Samer Ganam, Seema Biswas, Igor Waksman, Eli Kakiashvilli

**Affiliations:** 1 Department of Surgery A, Galilee Medical Center, Nahariya, Israel; 2 The Azrieli Faculty of Medicine, Bar-Ilan University, Tzfat, Israel; 3 Department of Urology, Galilee Medical Center, Nahariya, Israel; 4 Department of Surgery B, Galilee Medical Center, Nahariya, Israel

**Keywords:** Abdominal surgery, Re-laparotomy, Damage control surgery, Abdominal trauma, Missed injuries, War injuries

## Abstract

**Background:**

During the Syrian civil war, patients were initially treated on-site in Syria and later transferred to medical centers in Israel. Relevant details concerning the exact nature of injury and medical/surgical care received in Syria were unavailable to clinicians in Israel. Many of these patients required abdominal re-exploration for obvious or suspected problems related to their injury. Our aim is to present our approach to abdominal trauma patients who survived initial on-site surgery and needed subsequent abdominal re-exploration abroad, in our medical center.

**Methods:**

Clinical data from all medical records were retrospectively analyzed. Each patient underwent total body computerized tomography on arrival, revealing diverse multi-organ trauma. We divided the patient population who had abdominal trauma into 4 sub-groups according to the location in which abdominal surgical intervention was performed (abdominal surgery performed only in Syria, surgery in Syria and subsequent re-laparotomy in Israel, abdominal surgery only in Israel, and management of patients without abdominal surgical intervention). We focused on missed injuries and post-operative complications in the re-laparotomy sub-group.

**Results:**

By July 2018, 1331 trauma patients had been admitted to our hospital, of whom 236 had suffered abdominal trauma. Life-saving abdominal intervention was performed in 138 patients in Syria before arrival to our medical center.

A total of 79 patients underwent abdominal surgery in Israel, of whom 46 (33%) required re-laparotomy. The absence of any communication between the surgical teams across the border markedly affected our medical approach. Indications for re-exploration included severe peritoneal inflammation, neglected or overlooked abdominal foreign bodies, hemodynamic instability and intestinal fistula. Mortality occurred in 37/236 patients, with severe abdominal trauma as the main cause of fatality in 10 of them (4.2%), usually following urgent re-laparotomy.

**Conclusions:**

Lack of information about the circumstances of injury in an environment of catastrophe in Syria at the time and the absence of professional communication between the surgical teams across the border markedly dictated our medical approach. Our concerns were that some patients looked deceptively stable while others had potentially hidden injuries. We had no information on who had had definitive versus damage control surgery in Syria. The fact that re-operation was not performed by the same team responsible for initial abdominal intervention also posed major diagnostic challenges and warranted increased clinical suspicion and a change in our standard medical approach.

## Introduction

The horror and tragedy of the civil war in Syria is well-known and documented, including the fact that more than half a million people, mostly civilians, were killed. Beginning in 2013, some Syrian patients received definitive medical care in civilian hospitals across the border in Northern Israel. This scenario posed political and ethical dilemmas as Syria and Israel have been in a formal state of war for decades.

### Some key medical history data

With regard to the somewhat atypical management of war casualties described in this article, it is necessary to describe in brief the evolution of common medical doctrine so that the difficulties we experienced may be better understood. The modern medical approach and management of military trauma in Western armies, including in Israel, has evolved over the last 250 years. This doctrine dictates the relocation of dedicated and specialized medical units toward the frontlines of battlefields together with the provision of medical aid (definitive, if possible) as soon as possible, and accelerated transport of patients to trauma medical centers close to the frontline. The improvement in understanding and implementation of the principles of the modern medical approach to battlefield casualties has been clearly documented throughout history from the American Revolution and the American Civil War to World Wars I and II, the Korean, Vietnam and Afghanistan wars [1–9]. There has been a consistent and significant decrease in morbidity and mortality from combat trauma with increased understanding of the pathogenesis and management of diverse war injuries and their consequences [10–16]. The principles of modern military medicine have been fully adopted by the Israeli army [17].

### Medical circumstances during the Syrian civil war

In contrast, the conditions, circumstances and events pertaining to Syrian war casualties were different. Initial medical care patients received in Syria during the war was mostly sub-optimal as many hospitals were heavily damaged by massive bombing and there was a significant shortage of specialized medical personnel. Many of the wounded were from mass casualty incidents, so that, even when life-saving medical aid was accessible, aid was provided in austere environments that precluded efficient definitive medical care in most cases [18, 19]. There were no available survival data. Wounded patients who reached our hospital arrived typically days or even weeks after initial injury, often with hidden, overlooked or undertreated injuries. Thus, every patient presented a medical conundrum.

### Decision making concerning abdominal re-intervention

Due to lack of any communication across the border between the two countries, we admitted wounded patients without any accompanying medical information about the causes of injury, complications and interventions performed in Syria. We had no documentation of the nature of surgery performed in Syria – whether the procedure was that of damage control or definitive. No radiological or laboratory test results were available to us. The admission of critically injured Syrian patients under such circumstances created a major clinical dilemma. The question was: with no available history from the patient and the urgent need for organ support, would deferring abdominal surgery result in missed injury? Obviously, definitive surgery in Syria was not feasible in patients with unfavorable physiology resulting in hypothermia, metabolic acidosis and coagulopathy. Current military medical literature advocates that re-laparotomy be performed by the surgeons involved in the prior abdominal procedure. Our circumstances called for a different consideration in terms of indication and urgency [20–24].

The aim of our study was to summarize our cumulative medical experience specifically regarding re-laparotomy in patients who had already undergone initial surgery in Syria. We researched indications for re-laparotomy and prognostic parameters that might have predicted clinical deterioration with the aim of changing the strategic mode of care applicable to patients presenting under similar circumstances elsewhere.

## Methods

The study was approved by the institutional Ethics Committee (Helsinki). All methodology was in accordance with Ethics Committee instructions – in particular, the anonymimity of patient data. Researchers worked from coded data and data identifying patients were meticulously removed.

On their arrival, each patient was guided immediately into the emergency department for primary assessment and stabilization. Apart from a few patients who needed immediate surgery, each patient underwent total body computerized tomography on arrival in addition to full physical examination to exclude unexpected abnormal findings. For our study, all medical charts, medical documentation from the emergency room, operating rooms, and departments of surgery and intensive care were meticulously examined by experienced senior surgeons. All the data relating to type of injury, laboratory and imaging findings, indications for abdominal surgery, intra-operative findings and surgical reports, time from injury to arrival in our hospital, and injury severity score were uploaded into an Excel file for evaluation and statistical analysis.

### Statistical analysis

IBM – SPSS statistic software version 25 was used for statistical analysis. Quantitative variables were described using means and standard deviation. Qualitative variables were described using frequencies and percentages. Quantitative variables between sub-groups were compared using the independent sample t-test or the Wilcoxon rank sum test. Comparison of ordinal data was done using the Wilcoxon rank sum test, while comparison of qualitative data was done using the Chi square test or alternatively using Fisher’s exact test (when expectancy < 5). A *p*–value equal to or less than 0.05 was considered statistically significant.

We used univariate and multivariate logistic regression analysis to evaluate parameters related to mortality due to abdominal trauma. We report the R-square measure, *p*-value, odds ratio (OR) and 95% confidence interval (CI). The variables that were found to be significant in univariate analysis (*p*-value less than or equal to 10%) were chosen for inclusion in the multivariate model using the backward selection method.

## Results

Between the years 2013 and 2018, 1935 casualties of the Syrian civil war were treated in Galilee Medical Center, Nahariya, Israel. Of these patients, 1331 had war injuries. Abdominal involvement was noted in 236 patients (constituting our study group), with 138 patients previously operated in Syria. The main causes of abdominal trauma included blast injuries in 7%, shrapnel injuries in 45%, gunshot wounds in 40%, and combined injuries in 8%.

The time elapsed between injury and admission to our facility was 1–14 days in 77%. For 149 patients, arrival was within the first 7 days of injury. Twelve percent of patients arrived between 14 days and 3 months after injury. Eleven percent of patients arrived between 3 months and 3 years after injury.

Seventy-nine patients underwent abdominal surgery in Israel. In 46 of those 79 patients, surgery was re-laparotomy after previous abdominal surgery in Syria (46/138, 33.3%). Thirty two of 79 patients underwent primary laparotomy in Israel.

To better interpret the data, we divided our study group (236 patients) into four sub-groups: 1) patients who underwent abdominal surgery only in Syria – 93 patients; 2) patients who underwent abdominal surgery in both Syria and Israel—re-laparotomy—46 patients; 3) patients who underwent primary surgery in Israel – 32 patients; and 4) patients managed without any abdominal surgical intervention—65 patients. The mean age in the study group was 25.9 ± 9.4 years (range 3 to 61 years). There was no significant age difference among the four sub-groups (*p* = 0.12, Anova test). Twenty-five patients were children (3 to 16 years)—all equally distributed among the four sub-groups.

Of 52 patients operated on the day of arrival, 22 procedures were re-laparotomy (sub-group 2, 47.8%). The remaining 30 of 52 patients operated on the first day fell into sub-group 3 (30/32, 94%, *p* = 0.001, Chi square test). In 20 of 52 patients abdominal surgery was urgent, within two hours of arrival to our hospital. Of these 20 patients, 12 belonged to sub-group 3 (12/32, 37.5%), and 8 belonged to sub-group 2 (8/46, 17%, *p* = 0.042, Chi square test).

With regard to total body CT findings on arrival in our four sub-groups: brain injury rates were significantly higher in sub-groups 3 and 4—who had not undergone abdominal surgery in Syria—16/139 (11.5%) brain injury patients in groups 1 and 2 versus 28/97 (28.9%) in groups 3 and 4 (*p* = 0.001, Chi square test). It may be assumed that under the extreme conditions of war, fewer abdominal surgeries were performed whenever severe head injury was involved. The distribution of intestinal injuries (duodenum, rectum, large and small intestine) was significantly higher in sub-group 2 (re-laparotomy) in relation to other sub-groups, without taking into account associated trauma (27/46 patients, 58.7%, *p* = 0.001, Chi square test). The remaining injuries (liver, spleen, stomach, pancreas, diaphragm) were equally distributed between all sub-groups (*p* = 0.11 to *p* = 0.52, Fisher exact test).

Focusing on the re-laparotomy sub-group (sub-group 2), there were 46 patients, 40 of whom were male. The mean age was 26 years (range 9–55 years). Injury types included shrapnel (22 patients), gunshot [16], blast [5], shrapnel and gunshot [1] and unknown [2]. The indications for re-laparotomy were urgent in 39 patients, and planned in 7. Planned re-laparotomies for reconstructive procedures were scheduled electively leaving sufficient time for primary repairs to be fully healed. Indications for planned re-laparotomy included formation of loop colostomy (following abdominal surgery in Syria) for severe spine injury resulting in paraplegia in two patients, and closure of colostomy/ileostomy (intestinal re-anastomosis) following abdominal surgery in Israel in five patients. The time elapsed from the injury to arrival to our facility was within 24 h (3 patients), after 24 h ( 4 patients), 2 days (2 patients), 3 days (5 patients), 4 days (4 patients), 5 days (2 patients), 6 days (1 patient), 7 days (4 patients), 1 to 4 weeks (7 patients).

Urgent re-laparotomy was performed in 22 patients on the day of arrival. In 8 of these patients, re-laparotomy was performed within the first two hours of arrival. In 3 patients re-laparotomy was performed on day 2, and the remaining re-laparotomies were done on days 3, 4 and 5 (one patient on each day), day 6 (2 patients), and the others up to day 65. The main indications for emergent re-laparotomies were missed injuries in 19 patients (19/39, 48.7%) and complications of previous surgery in 22 patients, including 8 patients who had a combination of missed injury and post-surgical complications. Other miscellaneous indications were noted in six patients. Missed injuries and complications following previous abdominal surgery and miscellaneous indications requiring urgent re-laparotomy are detailed in Table 1. Without re-intervention these patients were likely to develop peritonitis, abscess formation, sepsis and hemodynamic instability.

**Table 1:** Indications for urgent abdominal re-operation including missed injuries, complications of previous surgery and other miscellaneous indications (39 patients). The table includes 6 cases with a combination of missed injuries and complications after previous abdominal surgery

	Cases (%)
**Missed injuries** (19/39 patients, 48.7%)	
Rectal perforation	2 (5.1)
Laceration of left large colon	5 (12.8)
Laceration of ureter	1 (2.6)
Diaphragmatic laceration, leading to traumatic hernia	2 (5.1)
Uncontrolled hepatic hemorrhage	1 (2.6)
Shrapnel injury to IVC	1 (2.6)
Gastric perforation, leading to sepsis and hemodynamic instability	1 (2.6)
Segmental intestinal necrosis	1 (2.6)
Gallbladder perforation	1 (2.6)
Colo-cutaneous fistula	1 (2.6)
Perforation of right colon	1 (2.6)
Recto-urinary bladder fistula	1 (2.6)
Recto-vaginal fistula	1 (2.6)
Small-bowel to retro-peritoneum fistula	1 (2.6)
**Complications following previous abdominal surgery** (22 patients)	
Failure of anastomosis of colon (ischemic necrosis)	2 (5.1)
Failure of abdominal wall suturing leading to evisceration	3 (7.7)
Failure of anastomosis of small bowel (ischemic necrosis)	4 (10.3)
Segmental necrosis of small bowel	1(2.6)
Injury to femoral vessels	1(2.6)
Failure of gastric suturing	1 (2.6)
Failed splenic hemostasis	1 (2.6)
Post-operative internal hernia	2 (5.1)
Failed suture of urinary bladder	1 (2.6)
Pancreatic necrosis	1 (2.6)
Abdominal compartment syndrome	1 (2.6)
Septic complications following surgery – peritoneal abscesses, peritonitis	3 (7.7)
Failed hepatic hemostasis	1 (2.6)
Failed diaphragmatic suture	1 (2.6)
**Miscellaneous indications**	
Removal of foreign body (medical pads deliberately left for emergent hemostasis)	1 (2.6)
Removal of neglected foreign bodies (medical pads)	4 (10.3)
Second-look exploratory laparotomy following multi-organ injury and hemodynamic instability	1 (2.6)

Clarification: Fecal drainage might be through surgical drains or via the wound, due to fecal leak from anastomotic dehiscence or rectal perforation (due to ischemia, shrapnel wound, etc.). It might be a missed diagnosis or post-operative complication or both

In 8 patients clinical presentation (rather than imaging evidence which did not necessarily reveal an urgent need for intervention) was the main factor in the decision to proceed to urgent re-laparotomy (Table 2). Abnormal computed tomography findings were the dominant factor in the decision for re-laparotomy in 14 cases. These patients did not have obvious peritoneal signs on clinical examination (Table 3). Combined abnormal clinical signs with abnormal CT findings were the deciding factor in 17 patients (Table 2). The range of surgical procedures performed in these 39 patients at urgent re-laparotomy are listed in Table 3.

**Table 2:** Assessing the role of clinical presentation and abdominal computed tomography as the dominant factors leading to re-laparotomy (39 patients)

	Number of cases (%)
**Clinical findings as the main factor leading to urgent re-laparotomy, without straightforward imaging evidence of intra-abdominal abnormalities—total 8 patients (number of cases (%)**	
Hemodynamic instability	3 (7.7)
Septic shock	1 (2.6)
Active bleeding (blood emerging from surgical drains)	1(2.6)
Fecal content emerging from surgical drains	2(5.1)
Acute abdomen	2(5.1)
Small bowel obstruction	1(2.6)
Abdominal wall evisceration	2(5.1)
**Abdominal computed tomography findings as the dominant factor leading to re-laparotomy (with a paucity of abnormal clinical signs – 14 patients)**	
Intra-abdominal free air and significant free fluid	4(10.6)
Intra-abdominal foreign body	3(7.7)
Intra-abdominal shrapnel in the vicinity of vital organs (IVC etc.)	2(5.1)
Inflammatory peritoneal involvement, fat opacity and fluid collections	4(10.3)
Suspected colorectal and urinary bladder injury	5(12.8)
Intestinal fistula to vagina and urinary bladder	3(7.7)
**Combined abnormal clinical presentation and abdominal CT modalities leading to urgent re-laparotomy – 17 patients**	
Hemodynamic instability and penetrating wound together with free abdominal gas and active vascular bleeding	1 (2.6)
Intra-abdominal fecal drainage together with extra-luminal gas and fluid collections, and fat opacity in the abdomen	4(10.3)
Abdominal tenderness and drainage of intestinal contents together with demonstration of traumatic diaphragmatic herniation (stomach, intestine)	1(2.6)
Abdominal tenderness and penetrating wound together with intra-peritoneal gas, fluid collections and foreign bodies	1(2.6)
Hemodynamic instability and abdominal compartment syndrome together with peritoneal free gas and fluids	1(2.6)
Sepsis, abdominal tenderness, fecal drainage together with peritoneal shrapnel, intestinal wall thickening, free fluid and gas	3(7.7)
Drainage of bile and worms from abdominal wound together with free gas and fluid and free peritoneal intestinal contrast	1(2.6)
Dirty drainage from pleural drain together with supra-hepatic free fluid with gas bubbles	1(2.6)
Open chest wound, severe abdominal wall wound and protruding pads, together with foreign body in the proximity of large intestine, and free abdominal gas	1(2.6)
Fever, tachycardia, gluteal dirty drainage together with gas bubbles and fluid anterior to psoas muscle	1(2.6)
Suspected intestinal fistula and fecal drainage together with intestinal obstruction, shrapnel and free peritoneal fluid and gas bubbles	2(5.1)

**Table 3:** Various surgical procedures at re-laparotomy in patients (46 subjects, second abdominal intervention in Israel)

	Number of cases (%)
Removal of abdominal foreign bodies	4 (8.7)
Repair of interruption of sutures of abdominal wall	3 (6.5)
Repair of intestinal fistula to vagina, urinary bladder and skin	2 (4.4)
Peritoneal lavage and debridement following peritonitis	29 (63)
Drainage of abscesses	8 (17.4)
Debridement of pancreatic necrosis	1 (2.8)
Revision and reconstruction of ileostomy	2 (4.4)
Suture of inferior vena cava	1 (2.2)
Hemostatic suturing of gastric vessels	1 (2.2)
Suture of Diaphragm	2 (4.4)
Various surgeries of the large intestine	15 (32.6)
Cholecystectomy	1 (2.2)
Re-hemostasis of hepatic and splenic bleeding	3 (6.5)
Resection of spleen	2 (4.4)
Various surgeries of the small intestine and duodenum	9 (19.6)
Resection of urinary bladder and creation of ileum conduit	1 (2.2)
Creation of colostomy or ileostomy	6 (13)
Gastro-intestinal anastomosis	1 (2.2)

We identified 3 patients during data analysis in whom earlier surgical re-intervention in our facility would likely have improved medical outcome. All 3 patients were alert and hemodynamically stable on arrival. In Syria they had undergone laparotomy for gun shot and shrapnel injuries. Peritoneal signs and fever were absent on arrival to our facility. In the first patient, abdominal CT revealed abnormal findings including large fluid collections containing free gas bubbles, free abdominal gas, fat opacification, suspected diaphragmatic injury and intra-peritoneal shrapnel. Re-laparotomy was performed 5 days after admission after fecal drainage was noted from a drain. This was a result of a missed sigmoid perforation and pelvic fecal collection. In the second patient, re-laparotomy was performed on day 13 following biliary peritonitis, sepsis and sub-diaphragmatic abscess (due to missed perforation of the gallbladder). In the third patient, relaparotomy was performed 15 days after arrival following gradual clinical deterioration and fecal drainage from the surgical wound (due to purulent peritonitis without obvious intestinal perforation).

Thirty seven patients died after admission to our medical center. We were able to determine the time between injury and arrival to our hospital in only 21 of those 37 fatalities. Among them, 10 were admitted within 24 h of injury, six within 24–48 h, and three within 48–72 h. We were unable to draw significant conclusions regarding the association between delayed arrival time and fatality rate. Eighteen patients among the 37 who died (48.6%) had already undergone abdominal surgery in Syria and 12/37 patients had abdominal surgery in our hospital. In eight of those 12 patients (67%) surgery in our hospital was re-laparotomy after previous abdominal surgery in Syria.

Forty-four patients in our study group had severe brain injury (44/236, 18.6%). Nineteen of these patients died (19/44, 43.2%, *p* = 0.001, Fisher exact test). Mortality rate in the remaining patients without significant brain injury was 9% (18/192). Brain injury was a significant factor in fatality in our abdominal trauma study group. Abdominal trauma was the primary cause of death in 10 patients (mainly due to fecal peritonitis, septic shock and blood loss, and multi-organ trauma), resulting in a 10/236, 4.2% mortality rate. However, as patients had multiple organ injuries, assigning abdominal trauma as the primary cause of death was based on consensus among the researches after careful analysis of the data. All 10 patients who died had abdominal surgery, 2 operated only in Syria, 3 only in Israel and 5 re-laparotomy in Israel after initial surgery in Syria. Thus, 7/10 of the deceased had had abdominal surgery in Syria and 5/7 (71.4%) needed repeat abdominal surgical intervention in Israel (group 2).

The main causes of death were severe brain injury (8 patients), septic shock and peritonitis (3 patients), severe pulmonary trauma and sepsis (5 patients), multi-organ trauma (pulmonary, vascular and orthopedic) and multiorgan failure (15 patients), vascular and orthopedic trauma including gas gangrene and septic shock (1 patient), and multi-organ failure with hemorrhagic shock (5 patients).

Mean injury severity score (ISS) of the survivors was 23 ± 13 while ISS of patients who died was 41 ± 14 (*p* < 0.001, t-test). Mean ISS was not significantly different among the four sub-groups (*p* = 0.23, Fisher exact test and *p* = 0.16, Kruscal-Wallis test).

Following multivariate analysis using the backward method, five parameters were found to be associated with mortality due to abdominal causes: severe splenic injury (*p* = 0.019), intestinal trauma (*p* = 0.064), ISS above 50 (*p* = 0.021), AST above 200u (*p* = 0.012), and the need for urgent abdominal surgery within 24 h of arrival (*p* = 0.038). Hemodynamic instability was not included as it does not necessarily represent abdominal trauma as the main etiology within the setting of multiple-trauma.

## Discussion

We describe our experience of dealing with war wounded patients undergoing re-laparotomy. Although our study focuses on re-laparotomy, in order to draw meaningful conclusions about the management of these patients, the extraordinary circumstances of their injuries and treatment should be taken into account.

### A short overview

The Israeli attempt at medical assistance for casualties of the Syrian civil war was a unique situation that posed humanitarian dilemmas associated with ethical, moral, military and political aspects [25]. Those patients who survived the initial injury and delayed transfer actually represent a selected group of severely wounded patients, some with very complex medical problems. The medical approach to the treatment of these patients, and the life-saving link between the medical teams on both sides of the border was largely different from modern war surgery protocols. In this article, we do not concentrate on the humanitarian, moral and political aspects as these have already been discussed in the literature [1–9, 25–28].

### What dictated our medical approach

Many survivors who managed to cross the border had already undergone life-saving surgical procedures in Syria under extremely austere conditions. Those patients did not carry with them any documentation regarding their medical or surgical treatment. Even though they were able to communicate in Arabic with clinical staff in Israel who speak Arabic, the patients were traumatized by their experiences, found themselves in the bewildering and high-tech environment of a well-equipped modern hospital with multiple departments and staff, and arrived with no relatives or friends who might help, orient, support or comfort them**.** Consequently, patients needed emotional support from hospital staff.

In addition, we assumed that medical staff in Syria were working with less sophisticated, inadequate, or no equipment, including, obviously, modern imaging modalities. Under these circumstances we adopted a medical approach of meticulous clinical re-evaluation in our facility with total-body computerized tomography that, not infrequently, revealed unexpected intra-abdominal findings [29]. This approach resulted in abdominal surgery even in patients who arrived in a hemodynamically stable condition after surgical intervention in Syria.

Abdominal surgical intervention performed in the battlefield or under dire circumstances did not preclude the need for repeated surgical intervention in our medical center (sometimes urgent). As we speculate that most surgeries abroad were life-saving and done under extreme conditions, we did not approach patients as having undergone definitive surgical solutions in most cases (most of our abdominal surgeries were done on the first day of admission). We assumed that abdominal trauma in those cases was so severe, that only life-saving procedures could have been done in Syria, and repeat surgery was indeed necessary in many situations, and, in some cases, obligatory. All the above reflect the significantly high proportion of re-laparotomies in our study (33.3%), undertaken mainly due to missed injuries and post-operative complications.

As expected, the most severely injured patients had missed abdominal injuries with additional risk factors (high ISS, multi-organ trauma and multiple organ failure) associated with severe morbidity [30, 31]. Comparing our re-laparotomy sub-group to the others showed that the rate of abdominal interventions was statistically higher, reflecting increased intestinal injury. This again emphasized that recent previous abdominal surgery was not a guarantee of definitive treatment.

There were pitfalls in our decision-making strategy. We delayed re-laparotomy in 3 patients, misled by their benign clinical presentation while imaging modalities revealed the need for immediate abdominal intervention. This stresses the high grade of suspicion needed to exclude missed injuries and make definitive diagnoses. Once again, our diagnosis and treatment algorithms were adapted to the unique circumstances of these patients.

As abdominal trauma was usually associated with multiple organ injuries, it should be noted that severe brain injury significantly influenced mortality rate in all sub-groups. We also hypothesized that the presence of severe brain injury reduced the rate of abdominal surgical intervention in Syria where insufficient resources precluded intervention in the austere environment.

### Mortality due to abdominal trauma in this context

The mortality rate primarily due to abdominal trauma (in association with severe comorbidities) should be highlighted. Analysis of the 37 patients revealed 10 who died as a result of abdominal trauma. All 10 underwent abdominal surgery, including five who had undergone surgery in Syria and subsequent emergency re-laparotomy in Israel (5/7, 71.4%), stressing again the severity of trauma in these cases resulting in a high fatality rate.

Following univariate and multivariate logistic regression analysis, we attempted to look for parameters that might reveal an association with mortality due to abdominal trauma in a multiple trauma scenario. In practice, however, it was not our intention to rely solely on those parameters as the complexity and interplay of many medical and other factors contributed to the clinical picture and decision making in these patients.

### Current and relevant literature perspective

Studies regarding damage control resuscitation and re-laparotomy for trauma have outlined the approach to multiple-trauma toward favorable outcomes and increased survival [32]. The approach includes immediate surgical intervention, limited intravenous crystalloid infusion, higher dose plasma and platelet infusion, and permissive hypotension. A recent article from a hospital in Damascus, Syria, reported outcomes in patients with penetrating abdominal injuries during the Syrian civil war [33]. Re-laparotomies were not reported, delayed transportation to hospital was not mentioned, and the decision for abdominal intervention was based mainly on clinical evaluation. Studies emphasize the importance of improved outcome of urgent and planned reoperation in patients with severe trauma [20, 21, 24, 34–36]. These studies show the central importance of the surgeon responsible for initial intervention and actively involved in the subsequent ongoing care of the patient and decisions for reoperation in reducing complication and mortality rates. Intrabdominal sepsis is a key factor, and early identification and intervention are crucial [22, 23]. The published rate of re-laparotomy ranges from 1 to 21%—higher in trauma patients [21–23, 34–38]. These studies are in contrast to the unique situation in which we looked after patients initially operated in Syria. It should be stressed that all decisions relied on clinical priorities only and each patient was given the best of medical, social and psychological care.

### Potential solutions

For nations between which exists a state of war, international humanitarian law, basic medical ethics and compassion form the foundation of medical care. Experienced high-quality surgical care is essential to outcome [39]. Senior clinicians and trainees in Israeli district civilian hospitals are acutely familiar with the physical, mental and social consequences of conflict. While six national trauma centers concentrate expertise in trauma in Israel, trauma and mass casualty management constitute a substantial component of emergency surgical training and all hospitals are equipped with state-of-the-art imaging modalities. This mitigated limitations in the sharing of patient information from Syria—where it is possible that the clinicians providing first-line care were themselves the target of hostilities within Syria [40]. Little is known about the conditions the first line clinical care providers in Syria were working under, but it is doubtful that they had access to radiological facilities [41]. In a few instances patients were able to give complete histories but for some patients any account of the mechanism of injury was associated with active conflict and not information they necessary wanted to recall or share. Some patients were in no physical condition to share information (unconscious, shocked, weak and disoriented). Thus, reliance on thorough clinical and radiological assessment was paramount, as was the need for vigilance and careful scrutiny of the response to treatment. Departmental social workers played a crucial role in the social and practical support patients required. Psychological and psychiatric support were also available. On discharge a comprehensive account of treatment was written in English for clinical staff or family receiving the patients and providing their ongoing care or support in Syria [42].

### Study limitations

The main limitation of our study lies in our inability to obtain enough data about the circumstances of injury and initial treatment. However, this is exactly what made the situation so unusual and exactly why we present here our decision making strategies.

## Conclusion

A strong index of clinical suspicion is warranted when handling patients in such exceptional, tragic and unusual scenarios. Each patient should be considered as potentially harboring an abdominal catastrophe requiring urgent abdominal exploration even when abdominal surgery has already been performed and regardless of initial impressions of clinical stability.

## Data Availability

Data is available from Prof Amitai Bickel but patient identifying data will not be shared as anonymization was crucial and data were stored (as per institutional ethics committee guidelines) as coded data.
